# Neuro-Vulnerability in Energy Metabolism Regulation: A Comprehensive Narrative Review

**DOI:** 10.3390/nu15143106

**Published:** 2023-07-11

**Authors:** Vicente Javier Clemente-Suárez, Ana Isabel Beltrán-Velasco, Laura Redondo-Flórez, Alexandra Martín-Rodríguez, Rodrigo Yáñez-Sepúlveda, José Francisco Tornero-Aguilera

**Affiliations:** 1 Faculty of Sports Sciences, Universidad Europea de Madrid, Tajo Street, s/n, 28670 Madrid, Spain; 2 Grupo de Investigación en Cultura, Educación y Sociedad, Universidad de la Costa, Barranquilla 080002, Colombia; 3 Psychology Department, Faculty of Life and Natural Sciences, Nebrija University, 28240 Madrid, Spain; 4 Department of Health Sciences, Faculty of Biomedical and Health Sciences, Universidad Europea de Madrid, Tajo Street s/n, 28670 Madrid, Spain; 5 Faculty of Education and Social Sciences, Universidad Andres Bello, Viña del Mar 2520000, Chile

**Keywords:** neuro-vulnerability, energy metabolism, metabolic disorders, neurobiological mechanisms, neuroendocrine interactions, neuroinflammation, neuroimaging techniques, eating disorders, obesity

## Abstract

This comprehensive narrative review explores the concept of neuro-vulnerability in energy metabolism regulation and its implications for metabolic disorders. The review highlights the complex interactions among the neural, hormonal, and metabolic pathways involved in the regulation of energy metabolism. The key topics discussed include the role of organs, hormones, and neural circuits in maintaining metabolic balance. The review investigates the association between neuro-vulnerability and metabolic disorders, such as obesity, insulin resistance, and eating disorders, considering genetic, epigenetic, and environmental factors that influence neuro-vulnerability and subsequent metabolic dysregulation. Neuroendocrine interactions and the neural regulation of food intake and energy expenditure are examined, with a focus on the impact of neuro-vulnerability on appetite dysregulation and altered energy expenditure. The role of neuroinflammation in metabolic health and neuro-vulnerability is discussed, emphasizing the bidirectional relationship between metabolic dysregulation and neuroinflammatory processes. This review also evaluates the use of neuroimaging techniques in studying neuro-vulnerability and their potential applications in clinical settings. Furthermore, the association between neuro-vulnerability and eating disorders, as well as its contribution to obesity, is examined. Potential therapeutic interventions targeting neuro-vulnerability, including pharmacological treatments and lifestyle modifications, are reviewed. In conclusion, understanding the concept of neuro-vulnerability in energy metabolism regulation is crucial for addressing metabolic disorders. This review provides valuable insights into the underlying neurobiological mechanisms and their implications for metabolic health. Targeting neuro-vulnerability holds promise for developing innovative strategies in the prevention and treatment of metabolic disorders, ultimately improving metabolic health outcomes.

## 1. Introduction

The brain is a highly energy-demanding organ that relies on the precise regulation of energy metabolism for optimal neuronal function and overall brain health. Disruptions in this delicate balance can lead to neuro-vulnerability and contribute to the development of various neurological disorders [1]. This comprehensive narrative review aims to provide an in-depth analysis of the neuro-vulnerability associated with energy metabolism dysregulation. It explores the underlying neurobiological basis, neuroendocrine interactions, neural regulation of food intake and energy expenditure, neuroinflammation, neuroimaging techniques, and their implications in eating disorders and obesity. Additionally, the review discusses therapeutic strategies for modulating neuro-vulnerability.

### 1.1. Regulation of Brain Metabolism

The human brain relies on the precise regulation of energy metabolism to meet its high metabolic demands. Disruptions in this delicate balance can lead to neuro-vulnerability and contribute to the development of various brain disorders [2]. Impaired glucose metabolism and compromised glucose uptake can result in energy deficits within the brain, compromising neuronal signaling and synaptic plasticity. Furthermore, disturbances in mitochondrial function, including reduced ATP production and increased generation of reactive oxygen species, contribute to energy metabolism dysregulation and neuronal vulnerability [3]. Dysregulated lipid metabolism, characterized by elevated levels of free fatty acids, can induce neuroinflammation, impair insulin signaling, and disrupt neuronal circuitry, exacerbating neuro-vulnerability [4].

Neuroendocrine interactions involving hormones such as leptin, insulin, ghrelin, and glucagon-like peptide-1 (GLP-1), play a crucial role in energy metabolism regulation [5]. Dysregulation of these hormonal signals can disrupt the energy balance and contribute to neuro-vulnerability. Additionally, the neural regulation of food intake and energy expenditure, particularly involving the complex interplay between the hypothalamic and brainstem circuits in appetite control, may contribute to a vulnerability to eating disorders and obesity. Eating disorders, including anorexia nervosa, bulimia nervosa, and binge-eating disorder, involve altered neural circuitry that is related to reward processing, impulse control, and emotion regulation. Disturbances in energy metabolism regulation, neuropeptides involved in appetite regulation, and neurotransmitter abnormalities have been implicated in the neurobiology of eating disorders [6,7]. Understanding the neuro-vulnerability in these disorders is essential for developing targeted interventions that address both the psychological and metabolic aspects. Similarly, neuro-vulnerability has been identified as a contributing factor to the development and progression of obesity. Dysregulated energy metabolism, impaired appetite regulation, altered reward sensitivity, and disruptions in the neural circuits involved in food intake control and energy expenditure have been observed in individuals with obesity [8]. Neuroinflammation and adipose tissue-derived inflammatory factors further contribute to neuro-vulnerability in obesity. Understanding the neurobiological mechanisms underlying neuro-vulnerability in obesity can inform the development of therapeutic strategies aimed at modulating the neural pathways involved in appetite control, energy balance, and metabolic regulation to manage and prevent obesity-related complications [9]. Conversely, compromised energy metabolism can trigger neuroinflammatory responses, creating a detrimental cycle.

This review also examines the potential interventions targeting glucose metabolism, mitochondrial function, neuroinflammation, and neuroendocrine signaling [10]. These interventions hold promise for mitigating neuro-vulnerability and improving neurological outcomes. Additionally, this review emphasizes the importance of neuroimaging techniques, such as positron emission tomography (PET) and functional magnetic resonance imaging (fMRI), in studying energy metabolism dysregulation and its association with neurological disorders. By exploring the neurobiological basis, neuroendocrine interactions, neural regulation of food intake and energy expenditure, neuroinflammation, neuroimaging techniques, and their implications in eating disorders and obesity, this review sheds light on the intricate mechanisms underlying neuro-vulnerability.

In conclusion, understanding the complex mechanisms of energy metabolism dysregulation and its impact on neuro-vulnerability is crucial for unraveling the pathogenesis of brain disorders and developing effective therapeutic interventions. The comprehensive analysis of neurobiological factors, neuroendocrine interactions, neural regulation of food intake and energy expenditure, and the role of neuroinflammation and neuroimaging techniques provides valuable insights into this field of research.

### 1.2. Methodology of Search

By encompassing both primary and secondary sources, various scientific articles, bibliographic indexes, and databases were utilized, including PubMed, Scopus, Embase, Science Direct, Sports Discuss, ResearchGate, and the Web of Science. The search was conducted using MeSH-compliant keywords to ensure the relevance of the gathered literature. Keywords such as neurological AND energy metabolism, endocrine, regulation, inflammation, imaging, behavior, eating disorder, obesity, and therapy were employed. The search period was limited to articles published between 15 June 2003 and 15 June 2023 to ensure the currency and pertinence of the data included in this review.

To ensure the appropriateness of the studies included in the analysis, the authors of the present review meticulously examined the titles and abstracts of all retrieved manuscripts. Exclusion criteria were applied to filter out studies that utilized outdated data beyond the designated timeframe, studies with unrelated topics that did not align with the specific objectives of this study, and studies not written in the English language. Once the relevant studies were identified, the same team of review authors independently extracted information from the selected articles. This rigorous approach helps maintain the quality and reliability of the data included in this review.

Furthermore, collaborative discussions were conducted among the review authors to synthesize the findings and present the current narrative review. By pooling their expertise and insights, the review authors ensured a comprehensive analysis of the literature and provided a cohesive and informative narrative that addresses the specific objectives of the study.

## 2. Neurobiological Basis of Energy Metabolism

The neurobiological basis of energy metabolism involves the intricate processes by which the brain regulates and utilizes energy for its proper functioning. Glucose is the primary energy substrate, and its metabolism is crucial for neuronal processes. Astrocytes play a vital role in maintaining energy homeostasis and providing metabolic support to neurons. Other metabolites, such as lactate, are under the focus of research, too [11]. Understanding the mechanisms underlying energy metabolism in the brain is essential for comprehending brain function and developing strategies to address neurological disorders related to energy metabolism dysregulation.

### 2.1. Metabolic Localization of Glycogen in the Brain

Glycogen plays a crucial role as the primary glucose storage form in human tissues, with diverse functions depending on its location. The liver regulates blood glucose levels by converting glycogen into glucose, which is then released into the circulation during periods of low blood glucose. Skeletal muscles utilize glycogen through glycolysis to meet increased energy demands, particularly during intense physical exercise. While glycogen levels in the brain are lower compared to the liver or muscles, its presence underscores its essential role in supporting neuronal activity. Astrocytes, found in the brain, are actively involved in neuronal metabolic processes, generating lactate and activating glycolysis and glycogen metabolism [11]. However, the extent to which astrocytes consume energy derived from glycogen themselves, rather than providing it to neurons as lactate, is still a subject of debate [12].

In terms of localization in the brain, glycogen exhibits specificity, primarily residing in astrocytes, although it has also been observed in embryonic neurons. However, the variations in glycogen levels among different astrocyte subtypes, distinguished by their morphology and functional characteristics, are not well understood [13]. Yet, glycogen distribution in the brain is not uniform, with higher concentrations observed in regions of dense synaptic activity, suggesting its involvement in synaptic transmission [14]. Gray matter generally contains approximately twice the concentration of glycogen compared to white matter. Significant levels of glycogen are found in regions such as the medulla oblongata, pons, cerebellum, hippocampus, hypothalamus, thalamus, cortex, and striatum [14].

Astrocyte end-feet, which express GLUT1 glucose transporters, extensively cover the capillary surfaces. These end-feet serve as communication sites with capillaries and participate in interactions with neurons and synaptic processes [15]. The collaboration between astrocytes and neurons in metabolic activities forms the foundation of the metabolic brain concept.

Enzymes within astrocytes facilitate glycogen metabolism in the brain, including glycogen phosphorylase (GP) and glycogen synthase (GYS). The brain isoform of GP is predominantly present in astrocytes but has also been identified in other cell types, like choroid plexus cells and ependymal cells. GYS, on the other hand, is found in neurons in the CNS [16]. The brain isoform of GYS shares a high degree of similarity with the muscle isoform and a lesser similarity with the hepatic isoform. GYS is widely distributed in the brain, prominently expressed in regions such as the hippocampus, cerebellum, and olfactory bulbs. It exists in both phosphorylated (inactive) and dephosphorylated (active) forms, allowing for the precise regulation of glycogen metabolism [17]. The interconversion between these forms is controlled by a family of phosphatases.

The brain’s energy metabolism is closely linked to compartmentalization, with distinct compartments in neurons and astrocytes characterized by specific conditions. Neurons possess synaptic vesicles exclusive to them [18]. The cytoplasm of brain cells exhibits heterogeneity, with localized high concentrations of metabolites, macromolecules, and ions. Mitochondria, responsible for energy production, also display heterogeneity [19]. Electron microscopy studies have shown the uneven distribution and varying potentials of astrocytic mitochondria within the same cell, indicating differences in their capacity for oxidative metabolism [20]. This suggests that specific mitochondria specialize in ATP production, while others fulfill auxiliary functions, such as the anaplerotic reactions involved in glutamine synthesis and its transport to neurons as precursors for neurotransmitters like glutamate and γ-aminobutyric acid (GABA). Mitochondria within astrocytes demonstrate dynamic behavior, constantly changing in numbers and forming networks [21].

### 2.2. Regulatory Mechanisms of Glycogen in the Brain

Glycogen metabolism is subject to complex regulation in various tissues, including the brain. Glucose is transported to brain cells through different glucose transporters, with astrocytes and oligodendrocytes utilizing GLUT1 and microglial cells utilizing GLUT5 [22]. Neurons, on the other hand, primarily rely on GLUT3 for glucose uptake. Glycogen synthesis and degradation can occur simultaneously and are tightly regulated processes.

Astrocytes, despite being non-excitable cells, play a crucial role in the absorption of excess potassium ions (K+) released by neurons during synaptic activity [23]. This process involves a mutual relationship between glycogenolysis (glycogen breakdown) and K+ uptake in astrocytes. Studies have shown that glycogen breakdown provides the necessary energy for K+ uptake in astrocyte cultures [24]. Halting glycogenolysis and inhibiting the activity of glycogen phosphorylase (GP) leads to a cessation of K+ ion uptake, indicating that astrocytes are primarily responsible for this activity [25].

The breakdown of glycogen in the brain is regulated by phosphorylation/dephosphorylation and allosteric mechanisms. Phosphorylation activates phosphorylase kinase (PK), which, in turn, phosphorylates GP, converting it from an inactive form (GPb) to an active phosphorylated form (GPa) [26]. PK is controlled by intracellular calcium levels, which can increase during periods of increased K+ uptake. The uptake of K+ also affects the intracellular pH and flow of carbohydrates, triggering the stimulation of adenylate cyclase (AC) and the production of cyclic adenosine monophosphate (cAMP). The subsequent binding of cAMP to protein kinase A (PKA) leads to the phosphorylation of PK. GPb can also be allosterically activated by AMP, which induces a conformational change in GPb, similar to the active GPa form. Glycogen also regulates the activity of AMP-activated protein kinase (AMPK), acting as an allosteric inhibitor of the kinase [27].

Glycogen synthase (GYS) is active in its dephosphorylated form (GYSa) and inactive when phosphorylated (GYSb). Covalent phosphorylation regulates the interconversion between GYSa and GYSb. Both GYS and GP can be simultaneously activated under certain conditions, leading to glycogen synthesis and breakdown [17]. The regulation of carbohydrate transport into the astrocytes is influenced by K+ ions and involves the Na^+^/HCO_3_^−^ cotransporter (NBC) [28]. Increased intracellular HCO_3_^−^ stimulates adenylate cyclase, cAMP production, and glycogen breakdown. Bicarbonate-induced glycogenolysis contributes to the activation of PKA and the PK/GP phosphorylation cascade [24].

Glucose in the brain can be oxidized through the pentose-phosphate pathway (PPP), which generates NADPH and 5-carbon sugars [29]. The PPP is crucial for lipid and nucleic acid synthesis, making it particularly important in the developing brain. Additionally, NADPH, derived from the PPP, is involved in metabolizing neurotransmitters and gliotransmitters, as well as in the regeneration of glutathione (GSH). Neuronal glycolysis is dependent on phosphofructokinase B3 (PFKB3), and its absence can lead to neuronal death under conditions of mitochondrial respiration inhibition [24]. Neurons can utilize alternative metabolic pathways, such as the PPP, for glucose oxidation.

Intracellular ascorbic acid has been shown to inhibit glucose uptake in neurons by inhibiting GLUT3, but it stimulates lactate transport through the same transporter [30]. This phenomenon, known as “the ascorbic acid metabolic switch”, alters the preference for energy substrates in neurons. Hormones, such as adrenaline, noradrenaline, and insulin, including insulin-like growth factors (IGF I, IGF II), also influence the brain’s glycogen levels by regulating glycogenolysis and glycogen synthesis. Additionally, the presence of ascorbic acid and its impact on glucose and lactate transport add another layer of complexity to the energy substrate preferences in the brain [31].

In conclusion, the regulation of glycogen metabolism and K+ uptake in the brain involves intricate mechanisms. Understanding these regulatory processes is crucial for comprehending brain metabolism and its implications in neurological conditions.

### 2.3. Lactate as a Metabolite?

Until recently, it was commonly accepted that neurons primarily generate energy through the oxidative metabolism of glucose, relying on the Krebs cycle and respiratory chain. However, emerging evidence indicates that neurons can efficiently utilize lactate as an energy source and even exhibit a preference for lactate when both glucose and lactate are available [32].

Notably, neurons lack the enzyme phosphofructokinase B3, which is involved in the glycolysis pathway for glucose, due to proteasomal degradation [33]. In contrast, astrocytes express high levels of this enzyme. Additionally, neurons have a slower rate of glycolysis due to their limited production of fructose-2,6-bisphosphate, a potent activator of phosphofructokinase, which is crucial for glycolysis [34]. Conversely, astrocytes have a higher rate of glycolysis, indicating their greater glycolytic capacity compared to neurons [35].

Interestingly, excessive activation of glycolysis in neurons can lead to oxidative stress and neuronal apoptosis, suggesting that neurons cannot sustain a high rate of glycolysis. On the other hand, increasing glucose concentration initiates the hexose monophosphate pathway (HMP) in neurons. Therefore, maintaining a delicate balance between the glycolytic pathway and the HMP cycle is crucial for meeting the energy demands of neurons while preserving their antioxidant status [24]. Utilizing lactate as a substrate may serve as an additional source of ATP production, apart from the glycolytic pathway, allowing neurons to conserve glucose for the HMP cycle and thereby protect themselves.

Astrocytes exhibit a high level of glycolytic activity and preferentially produce and release lactate, which can be further metabolized in the Krebs cycle. Previous assumptions suggested that astrocytes contribute only 5–15% of the brain’s energy needs [24]. However, recent evidence challenges this notion, indicating that the role of astrocytes in the brain’s energy metabolism processes might have been underestimated [36].

Studies have demonstrated that at rest, astrocytes in the rat brain are responsible for metabolizing approximately 50% of the glucose absorbed by the brain, with this percentage increasing during astrocyte activation [24]. This raises the question of how these findings reconcile with the fact that neurons, not astrocytes, have the highest energy demand. The transfer of energy substrates from astrocytes to neurons provides a plausible explanation for this apparent paradox [18].

Glutamatergic neuron activity leads to an increase in extracellular glutamate concentration, which is taken up by astrocytes through sodium-dependent glutamate transporters, elevating intracellular Na^+^ levels [37]. This triggers the activation of Na^+^/K^+^ ATPase, promoting glucose uptake and glycolysis in astrocytes. Consequently, lactate production increases and is released into the extracellular space [38]. Neurons then acquire lactate through monocarboxylate transporters (MCT) for subsequent metabolism. This concept of lactate transfer, first proposed by George Brooks, extends beyond astrocytes and neurons and encompasses various intracellular, intercellular, and intra-organ lactate transfers [24].

The uptake of glutamate by astrocytes enhances glucose utilization and lactate release. Intriguingly, when neuron and astrocyte cell cultures were exposed to glutamate stimulation, it resulted in increased glucose utilization in the astrocytes but rapidly inhibited glucose transport into the neurons [39]. Notably, this inhibition was more pronounced when lactate was present in the culture medium. The inhibition of glucose transport into neurons, and hence glycolysis by glutamate, necessitates the utilization of lactate as an alternative substrate by neurons.

Under these circumstances, glucose is utilized by neurons through the HMP pathway to generate NADP, which protects neurons against reactive oxygen species [40].

To expand on the topic, recent studies conducted in culture conditions have shown that hippocampal neurons experience a 50% reduction in synaptic transmission during medium glucose deprivation [41]. However, when glucose is supplied to the astrocyte syncytium, neuronal activity is restored. This restorative effect is similarly observed with the delivery of lactate, but it is not observed when a monocarboxylic acid transporter inhibitor, which transports lactate to neurons, is present [42]. This confirms that astrocytes metabolize glucose to lactate, which is subsequently utilized by neurons to maintain their synaptic activity. Numerous studies provide substantial support for the notion of net energy transfer from astrocytes to neurons in the form of lactate, particularly during glutamatergic transmission [11,18].

The transfer of lactate between astrocytes and neurons highlights the significance of glycolysis and lactate production in supporting neuronal activity. While astrocytes primarily engage in glycolysis and lactate generation, neurons primarily rely on oxidative metabolism. The transport of lactate across the blood-brain barrier is facilitated by the monocarboxylate transporter 1 (MCT1) [43]. Lactate uptake by the brain is influenced by the pH gradient between the blood and the brain, with a low pH leading to increased lactate uptake [44]. During exercise, when arterial lactate levels rise, the brain exhibits enhanced lactate uptake, particularly when a favorable H+ gradient is present [45].

Neuronal uptake of lactate primarily occurs through MCT2, a high-affinity isoform, whereas astrocytic uptake involves the expression of MCT1 and MCT4, which are low-affinity carriers with high transport capacities [46]. In the resting brain, neuronal lactate uptake is slower and approximately 60% saturated compared to astrocytic uptake. Intensive exercise, especially involving the legs, leads to increased cerebral lactate uptake [24]. Training can upregulate MCT expression, suggesting that both neurons and astrocytes may enhance their capacity for lactate transport in response to exercise.

During recovery from exercise, the lactate taken up by the brain is metabolized and not released into the blood or accumulated in the cerebrospinal fluid or brain tissue. Lactate may be utilized for synthesis or transported to other brain regions. The glucose-sparing effect of lactate may inhibit glycolysis through the conversion of lactate to pyruvate and subsequent oxidation, although it is still unclear if lactate is preferred over glucose. Lactate oxidation to pyruvate may be advantageous during low energy states, as it does not require ATP for activation [24].

In summary, intense exercise increases arterial lactate levels, decreases the pH, and enhances brain activation, all contributing to a higher cerebral lactate uptake. Lactate metabolism in the brain can account for up to 50% of glucose metabolism during exercise, supporting brain function. The lactate taken up by the brain is likely metabolized rather than released or accumulated. The coupling mechanisms between the neurons and astrocytes are essential for memory formation, with astrocytic glycogen breakdown and lactate release playing a crucial role in long-term memory. Lactate, but not glucose, can rescue amnesia caused by disruptions in astrocytic lactate transporters, highlighting the critical dependence of long-term memory on neuronal lactate uptake.

Thus, the neurobiological basis of energy metabolism involves intricate mechanisms that regulate glycogen metabolism and lactate utilization in the brain. Glycogen, primarily localized in astrocytes, serves as a vital source of energy to support neuronal activity. Astrocytes and neurons collaborate in metabolic processes, with astrocytes generating lactate and providing it to the neurons as an energy substrate. The regulation of glycogen metabolism involves enzymes, such as glycogen phosphorylase and glycogen synthase, as well as complex signaling pathways mediated by calcium levels, cyclic adenosine monophosphate (cAMP), and protein kinases. Furthermore, lactate has emerged as a significant metabolite in the brain, with evidence suggesting that neurons can efficiently utilize lactate as an energy source. Astrocytes exhibit high glycolytic activity and preferentially produce and release lactate, which is then taken up by neurons through monocarboxylate transporters. This lactate transfer from astrocytes to neurons is crucial for maintaining synaptic activity and supporting neuronal function. The regulation of lactate metabolism involves various transporters, pH gradients, and metabolic pathways, such as the hexose monophosphate pathway. Overall, understanding the neurobiological basis of energy metabolism provides valuable insights into brain function and its implications in neurological conditions.

## 3. Neuro-Vulnerability and Metabolic Dysregulation

The neuronal sensitivity of cell populations is not a field of study that has been widely addressed, although interest has been growing in recent years [47]. The genomic profile of neuronal populations, as well as their structure and functions, is now known, but neuronal adaptive responses to oxidative stress and other neurodegenerative factors are still a developing field of study [48,49].

Due to the blood-brain barrier, the CNS has traditionally been considered a protected system, as this barrier modulates the entry and exit of inflammatory cells and certain chemical mediators [50]. However, recent studies have shown that in healthy patients, reduced numbers of lymphocytes also appear in the cerebrospinal fluid (CSF). Proinflammatory cells or microglia cells that are involved in neuroinflammation and organic inflammatory propagation are present and actively participate in the neuronal sensitivity of acute and chronic pathologies [51,52]. This inflammatory response, which is expressed through the modulation of proinflammatory peripheral cells in the CNS, will favor an increase in the blood-brain barrier [52]. This is possible because the inflammatory mediators, such as prostaglandins and cytokines, induce the expression of these mediators and facilitate the release of harmful molecules to specialized brain cells [53,54].

Microglial cells have a CNS defense functionality and show a morphology that allows controlling the reception and propagation of inflammatory signals [55]. When microglia are activated, their functions are modified and the production of inflammatory cytokines and the presence of antigens as an immune response begins [56]. This is essential when neurological or neurodegenerative pathologies appear where the microglia are activated before a determined stimulus and a diversity of trophic factors and inflammatory mediators are secreted [57,58].

The same is true for astrocytes, as they can express these inflammatory mediators through the increased expression of GFAP or glial fibrillary acidic protein [59]. Astrocytes also secrete Th2-response stimulatory molecules, chemokines, and cytokines, in addition to secreting proteins and enzymes that are involved in neuronal cell damage. Among them are Matrix metalloproteinases (MMPs), the family of enzymes that degrade components of the extracellular matrix and are involved in tissue remodeling. The increased expression and activation of MMPs by astrocytes can contribute to neuronal cell damage [60], as well as phospholipases, since astrocytes can release phospholipases, such as phospholipase A2 (PLA2), which can hydrolyze phospholipids and generate bioactive lipid mediators, including prostaglandins and leukotrienes. These lipid mediators can induce inflammation and contribute to neuronal cell damage [61].

In this line, the accumulation of inflammatory cells and mediators is characterized by the presence of T lymphocytes, NK cells, and leukocytes that secrete cytokines that inflame the brain [62]. Cytokines are peptides that may differ in structure and genetics and act by binding to specific receptors on the cell surface. They are synthesized by different cells and can be classified into lymphokines and monokines, depending on whether they are secreted by macrophages or lymphocytes, and act on different neuronal populations [63]. The most important cytokines involved in the inflammatory processes are interleukins 1, 6, and 10 (IL-1, IL6, and IL-10), tumor necrosis factor-alpha or TNF-α, and growth factor-beta or TGF-β [64,65].

Thus, neuroinflammation, which is often observed in the presence of various pathologies, is associated with changes in the morphology of neuroglial cells and can contribute to tissue destruction [66]. This is closely related to the understanding of the bidirectional relationship expressed through the hypothalamic–pituitary–adrenal (HPA) axis that explains cellular neuroinflammation and the systemic low-grade proinflammatory state that is present when the neuronal vulnerability is identified in certain CNS populations [67,68].

In this line, when the organism presents metabolic alterations, abnormal chemical reactions are produced that hinder the correct functionality of the different organic systems involved in cellular function [69,70]. Among the most frequently diagnosed alterations at present are diabetes, obesity, arterial hypertension, and dyslipidemia [71]. All of them are related to sedentary lifestyles, unhealthy eating habits and high-risk factors for the appearance of cardiovascular pathologies, and although there may be predisposing genetic factors, it is possible to modulate the appearance and maintenance of these risk factors through simple actions in the daily care of the individual [72]. When a series of abnormal characteristics appear in the metabolism and the risk of suffering certain coronary pathologies increases, the physiopathology of the so-called metabolic syndrome or syndrome X is expressed in the organism [73]. These characteristics are increased fasting glycemia, abdominal obesity, increased levels of low-density lipoproteins or LDL in the blood and decreased levels of high-density lipoproteins or HDL, hyperinsulinemia [74].

This is a medical condition that predisposes the person to serious organic diseases, but recent studies have also found a direct relationship between metabolic disorders and mental pathologies [75]. The data collected indicate that, in patients with psychotic disorders, there is a prevalence of metabolic alterations of almost 50% (42.6% men/48.5% women), although it is not yet possible to determine whether the pharmacology used in this type of psychopathology is the cause of the alterations or whether, on the contrary, the metabolic abnormalities were already present before the use of the associated drugs [76].

This is a field of study whose interest has been increasing in recent years and which allows us to identify the synaptic mechanisms of action and the density of receptors to neurotransmitters that are modulated by insulin through different molecular mechanisms, inhibiting the reuptake of noradrenaline and increasing the reuptake of serotonin [77]. In addition, the amount of ionotropic amino-3-hydroxy-5-methyl-4-isoxazolepropionic acid or AMPA, long-term potentiation or LTP and the stimulation of GABA receptors on the postsynaptic membrane are regulated. On the other hand, recent studies indicate that excess insulin in the bloodstream modulates the neuronal metabolism of glucose and GLUT-4 transporters [78]. Animal model studies (neural insulin receptor knockout mice) have shown modifications in neuronal populations of the hypothalamus [79,80].

However, it is important to note that CNS cells maintain a balance of neurotrophic factor signaling aimed at inhibiting the inflammatory immune response of microglia [81]. When a pathogenic stimulus appears and the CNS mononuclear phagocytic cells are activated through cell membrane modification, a series of production and secretion responses of different metabolic enzymes are triggered [82]. This can lead to low-grade systemic inflammation and other pathological manifestations that hamper the homeostasis of microorganisms and the production of reactive oxygen species that promote oxidative stress in the organic cellular environment [83].

Although it is true that not all inflammatory processes are harmful, given the main function of these processes is to limit cell damage, oxidative mechanisms maintained over time favor neuronal damage and the chronification of inflammatory processes [84]. 

## 4. Neuroendocrine Interactions in Energy Metabolism

Historically, the study of energy balance has been closely related to the study of obesity. This has made possible an increase in the knowledge of this construct thanks to the initial research in animal models that suggested that the alterations presented in obesity and hyperphagia could be associated with specific brain structures, specifically the hypothalamic nuclei [85]. These structures were affected as the sites involved in appetite control and showed damage in the ventral, dorsomedial and paraventricular areas, as well as in the arcuate nucleus, and thus it was determined that they corresponded to the satiety center [86,87].

Currently, it is known that the cellular, endocrine, immune, and nervous regulatory systems form a unitary system that is maintained and interconnected by neuropeptides—small proteins that have a neurotransmitter function and that are also called polypeptides and that normally act through G protein-coupled receptors [88]. There are more than 100 identified neuropeptides with neurotransmitter functions that are associated with modulation of the sleep–wake cycle, feeding, pain and analgesia, learning, memory, and energy balance [89].

During the last decades, different neuropeptides located in the intestine, brain, and both organ systems have been discovered, all of them involved in the modulation of energy balance [90]. In addition, different cell cultures have been identified that present ample capacity for cell proliferation, as well as cytokines that are produced and secreted in the endothelial reticulum [91]. A clear example of the phenomenon of cell–cell binding by neuropeptides is energy regulation, also known as the diffuse neuroendocrine system (DENS) [92], which presents a wide range of specialized cells that make up such relevant structures as the adrenal medulla, the parathyroid glands, the paraganglia, or the endocrine pancreas, among others [93,94]. It is considered a division of the nervous system and has an essential organic homeostasis function [95].

In this line, neuropeptides have been discovered, which are secreted in the arcuate nucleus and which, thanks to their peripheral or intracranial innervations, send the appetite signal and are called orexigenic [96]. We find here the anabolic neuropeptide Y, composed of 36 amino acids, which is produced in the medulla oblongata, in the dorsomedial nucleus and the arcuate nucleus, and whose secretion to the paraventricular nucleus activates GABA or norepinephrine [97]. In contrast, the appetite regulation and shutdown signal are transmitted by anorexigenics through hypothalamic pathways. Leptin is one of these neuropeptides, secreted in the periphery, specifically by the ob gene of adipocytes, and whose function is the inhibition of neuropeptide Y [98]. It is a neuropeptide containing a peptide containing norepinephrine. It is a peptide containing 167 amino acids and is essential in understanding energy balance [99,100].

Leptin is secreted in the stomach, skeletal muscle, and adipose tissue and, together with cytokines, has a proliferative function on thymocytes [101]. Therefore, it is related to the level of fat in the body, mainly white fat tissue or WAT, and is associated with the energy balance and restriction of energy expenditure. When leptin levels during fasting are decreased, blood insulin levels are also reduced, although the sympathetic inhibition that has been shown in studies with animal models does not appear [102].

In humans, this decrease in leptin produces a reduction in plasma insulin, which leads to metabolic regulation due to a lack of energy and activates gluconeogenesis [103]. Both leptin and insulin produce adipose signals to regulate fat stability by activating receptors in the brain, although they reach the brain by saturation through the cerebrospinal fluid [103,104]. It is now known that leptin is a class of cytokine receptors and belongs to the interleukin-6 family [105]. Signals emitted by leptin receptor activation in skeletal muscle impact fatty acid metabolism and explain insulin sensitivity and glucose metabolism, utilizing the insulin receptor establishment phosphorylation circuits [106].

On the other hand, it is known that nutrients function as regulators of endocrine factors during the development of human beings from the fetal stage, even when it is unnecessary to activate appetite signals. Leptin levels during pregnancy regulate the placental weight and act as a sensor of fatty matter in the central nervous system and, thus, neuroendocrine actions [107,108]. The amount of leptin that reaches the brain will favor modifications in hormone levels that are essential for development, such as growth factors, thyroid hormones, or insulin. All this indicates that leptin functions as an adipostatic signal in the brain in direct relation to body fat and saturates the receptors located in the nuclei of the hypothalamus that regulates food intake [109].

Insulin also functions as an adipostatic signal in the brain and reaches the brain via saturable transport. Glucose stimulates insulin production in the pancreas through body fat levels [110]. Thus, when obesity is present and insulin resistance is established, the pancreas secretes more insulin and excess insulin results in increased fat storage in the adipocytes [111]. Disruption of insulin sensitivity is associated with diabetes, as well. In addition, insulin receptors are present in the same brain structures as leptin, mainly the arcuate nucleus [112]. Both signals are related to modifications in food intake, which are related to hypothalamic neuropeptides [113].

## 5. Neural Regulation of Food Intake and Satiety

It has been largely proposed by the previous literature how different molecules may modulate the satiety process and consequently regulate food intake. These molecules include motilin, ghrelin, glucagon-like peptide-1 (GLP-1), cholecystokinin, and leptin, among others.

In order to clarify how these different molecules may affect the neural regulation of food intake, it is important to explain the different phases in the cycle of hunger and satiation. Thus, a term in the literature has been proposed as MMC (migrating motor complex), which supposes three different phases. The first one, phase I, in where no contractility exists. In the second one, phase II, contractility starts to become evident, and its frequency and amplitude reach their upper levels. Finally, there is a last step, phase III, in which the maximum levels of contraction occur, and subsequently, the cycle restarts again [114,115]. Thus, the molecule that seems to be most important in MMC regulation is motilin, which is formed in the small intestine and causes gastric contraction [116,117]. In this line, several researchers pointed out how motilin values oscillate depending on the MMC phases, reaching the maximum gastric levels before phase III [118,119,120]. 

Another important molecule in the neural regulation of food intake is ghrelin. It is a hormone synthesized in enteroendocrine cells of the gastrointestinal tract, and it is known for its capability of modulating appetite [121,122], as it sends different indications from the gastrointestinal tract to the brain, more specifically to brain nuclei, modulating eating behavior, increasing food intake [122,123]. In this line, the peripheral ghrelin pathways ascend from the vagus nerve to the nucleus of the solitary tract, which is the most important sensory nucleus that collects, among others, cardiovascular and visceral information. From the nucleus of the solitary tract, ghrelin signals climb to the hypothalamic arcuate nucleus through noradrenergic projections synapsis. In the hypothalamus, ghrelin stimulates noradrenergic neurons by way of α1- and β2-adrenergic receptor activation, leading to feeding [124]. Moreover, in the presence of an endogenous ligand for the growth hormone secretagogue receptor, ghrelin is able to enhance food intake as well as it has the ability to promote growth hormone liberation [125]. However, although ghrelin presents certain similarities with motilin, its levels do not oscillate with the different MMC states. In this line, in order to emulate phase III of MMC, it would be necessary during the presence of mealtimes, motilin, or motilin agonist administration [119,126,127,128]. Moreover, regarding the motilin and ghrelin connection in food intake regulation, recent research conducted in rodents proposed how ghrelin also promotes gastric contractions in vitro in the presence of small motilin doses, as well as motilin encouraging robust gastric contractions under ghrelin during its in vivo presence [117,129]. Ghrelin levels may be modulated by different eating conditions. For example, raised ghrelin levels were found in patients who were suffering from fasting, whereas those levels decreased after oral glucose intake [123]. Hence, ghrelin levels have been found significantly decreased in obese patients [130], and when weight loss occurred, these levels increased, and later they suffered another decrease when the patients suffered weight recuperation [131,132]. These results suggested how the modification in ghrelin levels may modulate different weight variations. 

Regarding glucagon-like-peptide-1 (GLP-1), it is a hormone produced by endocrine L-cells originating from the intestinal epithelial in reply to meal intake. GLP-1 enhances insulin secretion as well as reduces glucagon liberation, leading to a reduction in postprandial glucose and fasting. Concerning its activity in the gut, it has been related to a reduction in its motility, as well as to a reduction in acid secretion. Finally, it modulates food intake and controls appetite [133], as it reduces gastric emptying, inhibiting food intake [134]. It may be explained by its capability of activating brown fat as well as raising energy expenditure through sympathetic nervous system pathways activation [135]. Thus, GLP-1 may cross the blood-brain barrier, accessing the brain through passive diffusion. Then, it may activate the GLP-1 receptors in hypothalamic nuclei, leading to a reduction in weight [136]. In this line, the nucleus of the solitary tract also seems to play an important role in food intake regulation, as it has also been related to GLP-1 activity. Then, it presents neurons that express the proglucagon gene encoding GLP-1 as well as proglucagon-derived peptides. Then, recent studies conducted in murine models proposed how the activation of proglucagon gene-encoding neurons may decrease food consumption, metabolic rate, and glucose production [137]. Similar results were found by other researchers, in which a chemogenetic stimulation of the brainstem nucleus of the solitary tract of the proglucagon gene encoding GLP-1 decreased food intake in high-fat diet-fed mice [138]. GLP-1 involves satiation mechanisms triggering a reduction in food intake. In this line, it has been described how GLP-1 activates the vagus nerve, which in turn excites different sites, such as the nucleus of the solitary tract neurons. Subsequently, these solitary tract neurons activated the discharge of endogenous GLP-1 from their axons, which act in GLP-1 receptors, resulting in meal cessation [139,140,141].

Cholecystokinin is a hormone produced and liberated by the enteroendocrine cells of the duodenum. It is known for its capability to enhance enzyme and bile discharge, promoting digestion by a reduction in gastric emptying [142]. Furthermore, it also has been pointed out as a useful hormone that may modulate food intake, acting as a satiety signal that modulates the short-term control of food consumption [143,144,145]. Cholecystokinin involves satiety signaling that is also related to the pathways of the nucleus of the solitary tract, which it reaches via the vagal fibers, and consequently achieves paraventricular nuclei and nuclei of the ventromedial hypothalamus, constituting an autocrine signal mechanism, triggering the satiety effect [146,147].

Leptin is a hormone secreted by the adipose cells. It is known for its capability of reducing fat deposits in the adipocytes as well as reducing appetite [148]. The activity of leptin is mediated by its binding to its receptors, which are extensively expressed both in the brain and peripheral organs. These receptors have been largely described in previous literature due to their important activity in regulating food intake. One of the isoforms, leptin receptor ObRsa or short leptin receptor isoform, is extensively expressed in the brain-blood barrier and allows the pass of leptin to the brain. The other isoform of the receptor, called ObRb, or long leptin receptor isoform, is located in the hypothalamic nuclear groups and controls eating behavior and modulates body weight through signal transduction, primarily the signal transducer and activator of transcription (STAT), which has demonstrated its implication in energy homeostasis regulation [149,150,151]. Thus, it was described by previous researchers how the administration of leptin in patients who showed decreased leptin levels might inhibit food consumption, enhance satiety pathways, and control body weight [152].

## 6. Neural Control of Energy Expenditure

Energy expenditure is a highly regulated process that is crucial for maintaining energy balance in the organism. The neural control of energy expenditure plays a fundamental role in orchestrating metabolic processes and ensuring the efficient utilization of energy resources. 

Neural control of energy waste involves the activity of different organs, including the gastrointestinal tube, muscle, and adipose storage, such as white adipose tissue (WAT), brown adipose tissue (BAT), and various viscera, such as the liver and pancreas. As a result, the metabolism may be affected in its various stages, including production, depot, mobilization, and usage, enhancing energy source product development [153].

The neural control of energy waste occurs predominantly in the hypothalamus, which is the central axis for coordinating energy balance. More specifically, the regulation of energy expenditure occurs in a specific area, the arcuate nucleus, in which two types of neurons exist and have opposite functions. Firstly, the arcuate nucleus presents orexigenic neurons, which stimulate appetite and increase energy consumption. On the contrary, it also presents the anorexigenic neurons, which suppress appetite and enhance energy expenditure [154]. Thus, depending on the state of fasting or food intake, different hormones may be secreted, promoting the activity of those neurons. In this line, it has been pointed out by previous literature how leptin, which is released by adipose tissue, acts as a satiety signal and inhibits orexigenic neurons while stimulating anorexigenic neurons, whereas ghrelin, produced in the stomach, promotes hunger and stimulates orexigenic neurons [155]. Additionally, other hormones have been highlighted for their capability of modulating neural control of energy expenditure, such as cortisol, TSH, and insulin [156,157,158].

Regarding leptin, it has been highlighted by its pivotal role in the neural control of energy expenditure [159]. Hence, the previous literature suggested how leptin receptors are expressed in the arcuate nucleus in the hypothalamus, where they interact with different populations of neurons. Thus, by acting on specific receptors within the hypothalamus, leptin influences the appetite and energy balance regulation. In this line, when leptin activates anorexigenic neurons, they produce pro-opiomelanocortin (POMC), a polypeptide precursor of other proteins, which consequently triggers the release of alpha-melanocyte-stimulating hormone (α-MSH). Consequently, α-MSH acts on melanocortin receptors, suppressing appetite and increasing energy expenditure [160]. Nevertheless, when leptin activates orexigenic neurons, they produce neuropeptide Y (NPY) and agouti-related peptide (AgRP) [161,162], two substances involved in the orexigenic processes, as AgRP impedes α-MSH activity and NPY acts on the specific receptors increasing food intake [151,163,164,165]. 

Concerning ghrelin, it has been pointed out by its dual activity controlling neural expenditure waste [166,167]. In this line, it has been highlighted as an orexigenic substance, given it stimulates appetite and promotes food intake. Moreover, it has been related to thermogenesis processes regulation, and as a consequence, it has been related to energy metabolism modulation [168]. This modulation has been associated with the fact that ghrelin has been shown to stimulate the release of growth hormone (GH) in the pituitary gland. GH, in turn, has direct effects on energy metabolism by promoting lipolysis and the utilization of fatty acids as an energy source. In addition to its influence on GH release, ghrelin also interacts with other neurotransmitters and signaling systems in the brain that are involved in appetite control and metabolism. For instance, ghrelin has been observed to increase dopamine release in the brain areas associated with reward and motivation, leading to a heightened desire for food consumption and, consequently, an increase in energy expenditure [169]. Regarding ghrelin’s activity on thermogenesis, it has been proposed by recent literature how it may activate receptors in the hypothalamus, triggering the release of neuropeptides, including orexin and melanin-concentrating hormone, which have been implicated in the promotion of thermogenesis [170]. Moreover, ghrelin influences thermogenesis through its effects on brown adipose tissue (BAT), a specialized fat tissue that generates heat through the process of uncoupling protein 1 (UCP1)-mediated thermogenesis. Thus, recently it was proposed how ghrelin may enhance UCP1 expression and activity in BAT, thereby increasing energy expenditure and heat production [171,172].

With regard to TSH, it also has been pointed out by its activity, which modulates energy expenditure. TSH, also known as thyrotropin, is a hormone produced by the pituitary gland that plays a central role in the regulation of thyroid function. While its primary function is to stimulate the thyroid gland to produce thyroid hormones, TSH also exerts direct effects on energy expenditure and metabolism [173]. Then, when TSH stimulates the thyroid gland to produce thyroid hormones, it promotes thyroid hormone liberation. Hence, the thyroid hormone modulates metabolism through different organs, including the brain, WAT, BAT, skeletal muscle, liver, and pancreas. Most of the associated processes suppose modulation both in energy storage and expenditure [173]. Furthermore, TSH has been shown to affect energy expenditure through its actions on BAT. BAT is a specialized fat tissue that plays a crucial role in thermogenesis and energy dissipation. TSH receptors are expressed in BAT, and activation of these receptors has been found to stimulate thermogenesis and increase energy expenditure. This process is performed by UCP1 being present in the internal mitochondrial membrane of BAT. Thus, when an adrenergic discharge activates UCP1, it disperses energy as heat by deactivating oxidative phosphorylation [174]. Thus, when TSH activates thermogenesis, there is an increase in energy expenditure due to increased heat production and accelerated nutrient metabolism.

Regarding cortisol, it has been proposed by previous authors how it also may be related to the neural control of energy expenditure. Firstly, cortisol affects appetite and food intake regulation. High levels of cortisol have been associated with increased food intake, particularly a preference for energy-dense foods high in carbohydrates and fats [175,176,177,178]. This can lead to an increase in caloric intake and subsequently impact energy expenditure. Moreover, cortisol influences the neural pathways involved in energy homeostasis, particularly those related to the hypothalamus. Cortisol receptors are present in the key hypothalamic areas involved in appetite regulation, including the arcuate nucleus and the paraventricular nucleus, as mentioned above. Hence, cortisol can modulate the activity of these neurons by decreasing leptin’s sensitivity in orexigenic and anorexigenic neurons [179]. Subsequently, it could enhance AgRP and NPY activity [180], and as it was explained before, it could increase food intake through α-MSH inhibition due to AGRR activity, as well as by acting on specific receptors as a result of NPY activity.

## 7. Neuroinflammation and Metabolic Health

Over the past few decades, our understanding of CNS (central nervous system) inflammation has progressed. Whether the disruption to homeostasis originates within or externally, the result is the same: neuroinflammation. Direct and indirect immune-related neuroinflammation plays a role in the pathogenesis of many neurological illnesses, including those caused by trauma, tumors [181], metabolic abnormalities, toxins, infections, autoimmunity, development [182], and degeneration [183]. Brain infiltrates of innate and adaptive immune cells, which produce inflammatory mediators, occur in response to an infectious or noxious substance. Both resident and visiting cells and signals can act as mediators of inflammation. Thus, destructive cytokines, proteases, glutamate, free radicals, and glial cell activation are released when there is an overwhelming inflammatory response in the CNS [184]. Concretely, neuronal loss and an uptick in proinflammatory cytokines like tumor necrosis factor-alpha (TNF-α) and interleukin (IL)-1β, in addition to nitric oxide (NO) are characteristic of the neurodegenerative illnesses associated with abnormal conditions in the microglia [185]. Moreover, the central nervous system’s inflammatory response can also be caused by processes resulting from disorders of the central and peripheral nervous systems [183,186]. Likewise, a genetic predisposition [187], environmental factors [8], free radical production [188], excitotoxicity, neuroinflammation, disruption of the calcium regulation system, mitochondrial dysfunction [189,190], and the accumulation of unfolded proteins [191] can all play a role in the progression of neurodegenerative diseases.

**Figure 1:**
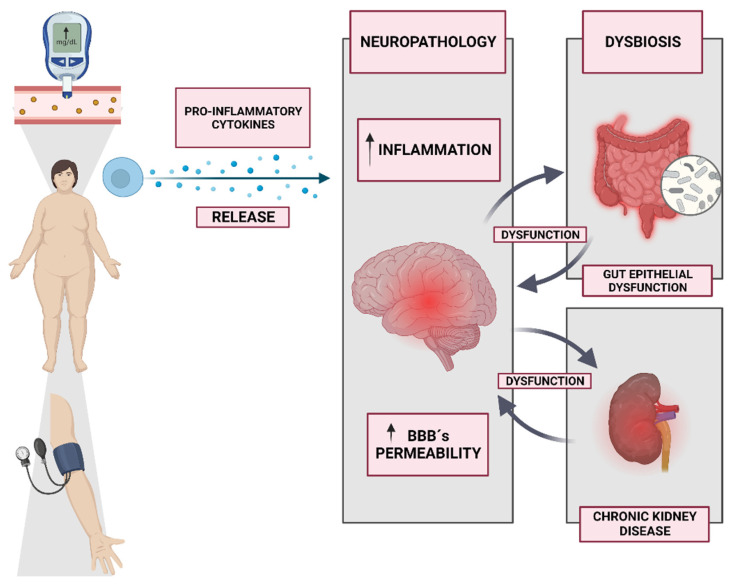
Metabolic complications causing neuroinflammation and the different processes affecting the kidney–brain and gut–brain axis.

Aging and metabolic illnesses, including obesity, hypertension, diabetes, depression, dementia, or brain injury, likely share common cellular and molecular processes that might set off neuroinflammation (Figure 1) [192]. Concretely, epidemiological studies have established a link between diabetes and an increased risk of cognitive decline among the elderly. Several mechanisms have been postulated to explain this association, including central insulin signaling, neurodegeneration, brain amyloidosis, and neuroinflammation, based on animal model research [193]. Concretely, in diabetes patients, hyperglycemia, insulin resistance and altered insulin signaling, neuroinflammation, cerebral microvascular injury, and accumulation of cerebral amyloid and tau proteins are among the postulated pathophysiological mechanisms underlying cognitive decline [193]. In observational studies, those with type 2 diabetes mellitus (T2DM) appear to be at a higher risk of developing Parkinson’s disease (PD), as well as experiencing rapid progression and a more severe phenotype of PD, with the effects possibly mediated by several common cellular pathways. By means of insulin dysregulation, amyloid aggregation, neuroinflammation, mitochondrial dysfunction, and altered synaptic plasticity, for instance, the insulin signaling pathway may be responsible for neurodegeneration [15]. Although few conclusive research studies have attempted to identify specific biomarkers, Ehtewish et al. showed that C-reactive protein, tau protein, brain-derived neurotrophic factor, advanced glycation end products, glycosylated hemoglobin, and adipokines should be considered in diabetes-related cognitive decrements [194]. Xu et al. also specified that mild cognitive impairment in type 2 diabetes patients is characterized by aging, peripheral circulating glycogen synthase kinase-3β (GSK-3 β) activation, expression of ApoE ε 4, and an increase in the olfactory score; the combination of these indicators can improve diagnostic accuracy [195].

In more complex diseases, however, it has been found that nutrition plays a role in the inflammatory processes that can harm the brain. Concretely, systemic inflammation caused by a high-fat diet raises the amounts of proinflammatory cytokines in the blood, which causes inflammation in the center of the body. These moving proinflammatory cytokines move into the brain and hypothalamus by crossing the blood-brain barrier (BBB). This also turns on Nuclear Factor κB (NF-κB) in the microglial cells in the hypothalamus, which causes inflammation and resistance to leptin in the hypothalamus (Figure 1) [196]. The hypothalamus is an important control center for many bodily activities, including energy balance, stress management, reproduction, and even cardiovascular health. Attention, learning, and many facets of memory and cognition are all intricately linked to many of these processes [197,198]. The paraventricular nucleus of the hypothalamus (PVN) is the epicenter of the hypothalamic–pituitary–adrenal axis (HPA), and its dysregulation is linked to memory loss [199]. High morning cortisol levels are strongly tied to the function of the HPA axis, which explains why depressed patients suffer from deficiencies in executive function and memory recall [200]. Specifically, Cope et al. demonstrated that the hippocampus of humans and animal models of Alzheimer’s disease (AD) showed signs of diet-induced microgliosis, which suggests a possible mechanistic link between obesity/type 2 diabetes and cognitive impairment/memory decline in this prevalent medical condition [201]. Thus, it has been shown that an excess of saturated fatty acids and simple carbohydrates in the diet is a known environmental risk factor for AD, but a comprehensive understanding of the interdependent processes through which such a diet may contribute to AD pathogenesis is lacking. The Western diet evokes metabolic changes [202], induction of obesity and adipose tissue inflammation, gut microbiota dysbiosis [203], and the acceleration of low-grade systemic inflammation, which causes impairment of the BBB and the development of neuroinflammation, which is accompanied by the accumulation of toxic amyloid [204].

Additionally, one of the most common health problems caused by diet, among other factors, is metabolic syndrome, which encompasses obesity and diabetes [205]. It causes widespread inflammation throughout the body, the full extent of which has yet to be discovered. Increased permeability of the BBB is a common consequence of the neuropathologies produced by metabolic syndrome (Figure 1) [206]. The BBB is critical for healthy brain function because it blocks harmful substances, immune cells, and pathogens from entering the brain. BBB disintegration, poor waste clearance, and increased infiltration of immune cells have all been linked to the local and systemic inflammation brought on by obesity and type 2 diabetes mellitus [207]. Depending on the region of the brain that is impacted, this might lead to hormone imbalance, heightened immunological sensitivity, or cognitive impairment due to glial and neuronal cell disruption [208].

Regarding the gut–brain axis, bidirectional communication between the gut microbiota and the gut epithelium is implicated in the regulation of autonomic nervous system activity and blood pressure control [209]. This dysfunction in the gut–brain axis, characterized by dysbiosis of the gut microbiota, gut epithelial dysfunction, and deranged input to the brain, contributes to the development of hypertension through various mechanisms, including the release of inflammatory mediators, metabolites, and bacteria into the circulation, as well as alterations in afferent information [210]. These processes ultimately lead to neuroinflammation and an imbalance in autonomic nervous system activity, resulting in increased blood pressure. Furthermore, this dysfunction in the gut–brain axis has a negative impact on gastrointestinal function and the composition of the gut microbiota, exacerbating the problem [211]. In relation to hypertension, Richards et al. [211] proposed that immunoglobulin A-coated bacteria originating in the gastrointestinal tract and having access to the brain may play a role. However, it is worth noting that minocycline, known for its anti-inflammatory and antimicrobial properties, is being evaluated as a potential antihypertensive agent targeting the gut–brain axis.

A recent review also specified that chronic kidney disease (CKD) also implies neurodegeneration [212]. Concretely, epidemiologic studies imply that people with CKD at any stage are more likely to acquire neuropsychiatric disorders, cognitive impairment, and dementia. The significant incidence of both symptomatic and subclinical ischemic cerebrovascular lesions explains this risk [213]. It refers to the clinical, metabolic, and hormonal abnormalities that accompany progressive kidney failure [214]. Each patient with uremic syndrome of CKD develops cognitive decline, encephalopathy, seizures, asterixis, myoclonus, restless limb syndrome, central pontine myelinolysis, stroke, extrapyramidal movement disorders, neuropathies, and myopathy being among them (Figure 1). Their pathogenic mechanisms are intricate and varied [212]. In fact, Corona et al. specified that this decline in cognitive function impairs the ability to differentiate between odors in CDK patients. It is unclear whether peritoneal dialysis and hemodialysis enhance olfactory deficits, whereas renal transplants have a significant beneficial effect [214].

Anti-inflammatory medications target various pathways, but because the picture is so complex, it is impossible to foresee what side effects they might induce [215]. Given that some inflammation is required, it is critical to pick the therapeutic targets that will influence the patient’s immune response in a safe manner [216]. Resolving metabolic memory may be one of the more important therapeutic options, as it causes ongoing inflammation even after the blood glucose levels are normalized [208,216]. Although some data exist, future research should focus on the impact of specific dietary components, such as fatty acids, cholesterol, and carbohydrates, in the advancement of pathogenesis, which implies neuroinflammation, such as AD. Yet, recent meta-analyses of randomized trials and observational studies have presented intriguing findings regarding the impact of reducing saturated fat intake on CVD and total mortality. These studies have indicated that limiting saturated fat intake may not yield significant beneficial effects in terms of reducing CVD and overall mortality. Conversely, they have revealed potential protective effects against stroke and have shown no increased risk of CVD associated with the consumption of whole-fat dairy or dark chocolate. These important findings challenge the traditional recommendations to restrict saturated fat intake and emphasize the need for a comprehensive reassessment of the relationship between saturated fat and cardiovascular health [204].

Future research should investigate the interaction of environmental and genetic risk factors. The epigenetic modifications that may be caused by environmental risk factors, such as high-fat diets, and the likelihood of their transmission to succeeding generations as disease-promoting vs. disease-protecting predispositions are particularly intriguing.

## 8. Neuroimaging Techniques in Studying Neuro-Vulnerability

Neuroimaging techniques play a crucial role in studying neuro-vulnerability, which refers to an individual’s susceptibility to developing neurological disorders or experiencing changes in brain function [217,218]. These techniques allow researchers to visualize and investigate the structure, function, and connectivity of the brain, providing valuable insights into the underlying mechanisms of neuro-vulnerability [219].

Magnetic resonance imaging (MRI) is a powerful medical imaging technique that uses a strong magnetic field and radio waves to generate detailed images of the internal structures of the body. In this line, MRI plays a crucial role in understanding clinical diagnoses and the treatment of neuro-vulnerability associated with neurodegenerative disease by providing valuable information about the structure and function of the nervous system [220]. Moreover, this technique can help identify structural abnormalities in the brain, such as tumors, vascular malformations, or developmental disorders, which can contribute to neuro-vulnerability [221]. By visualizing these structural changes, MRI helps in the early diagnosis and monitoring of these conditions, allowing for appropriate management and intervention [222].

Thus, Mei et al. [223] used MRI to observe the sex differences in clinical manifestations of males and females with major depressive disorder (MDD). The authors demonstrated that the pathophysiological and developmental mechanisms of MDD differ in male and female patients, which could lead to sex differences in the clinical manifestations of MDD. Furthermore, this has important relevance in the treatment of MDD, given that sex-specific targeted therapies can be developed. Regarding Parkinson’s disease (PD), which is characterized by the selective loss of dopaminergic neurons in the substantia nigra, He et al. [224] hypothesized that magnetic resonance imaging could help to differentiate the pathophysiology of this disease from other neurodegenerative diseases. However, the results showed that, although MRI could potentially provide useful disease state biomarkers for clinical trials in the preclinical and early stages of Parkinson’s disease, it is insufficient to reliably distinguish atypical Parkinsonian disorders or to monitor the disease’s progression in moderate to late-stage patients. Furthermore, Li et al. [225] showed that MRI could be used as a potential biomarker to diagnose preclinical Alzheimer’s disease (AD). In this line, Wang et al. [226] stated that MRI is currently the gold standard for the early diagnosis of AD, which is caused by atrophy of the medial temporal lobe.

Apart from MRI, new neuroimaging techniques, such as single photon emission computed tomography (SPECT) and positron emission tomography (PET), have been used for the diagnosis of neurodegenerative diseases. These techniques are a type of imaging technology that involves the use of a specific marker for localization and quantification to show the onset and development of diseases at the molecular level [227]. In terms of PD, the diagnosis was based on the clinical judgment of expert professionals without considering certain biomarkers with high sensitivity and specificity [228]. However, these techniques can be very useful when it comes to an early diagnosis through certain markers, such as the pre-synaptic dopamine transporter (DAT) [227]. Concretely, DAT allows us to evaluate the integrity of the nigrostriatal pathway in vivo, providing an objective measure of neuronal degeneration in PD [229]. However, SPECT has some type of technical limitations due to its hardware design, such as limited spatial resolution and high image noise [230]. For this reason, some researchers have developed algorithms to solve these problems. In this line, Shiiba et al. [231] developed a DAT-SPECT image-derived radiomics signature and reported that their model would be useful for the diagnosis of PD and would have the potential to provide robust diagnostics. In addition, SPECT has been used in the diagnosis of AD due to its potential to provide information on regional cerebral blood flow, which has been postulated as a determinant of this disease. According to this, Höller et al. [232] reported that the quantitative analytical combination of data reported by the electroencephalography and SPECT markers was effective in differentiating AD from other diseases.

Therefore, these techniques can be very useful for the early detection of this type of neurodegenerative disease. However, more in-depth research is needed to provide clinical help and explore the pathophysiological mechanism of these diseases.

## 9. Neuro-Vulnerability and Eating Disorders

While eating disorders (ED) have psychological and social components, research suggests that they also involve biological and neurological factors. In this line, it has been postulated that eating disorders cause a series of behavioral changes and brain plasticity like those of obesity (Schmidt). In this sense, some research has reported that some temperament disorders and personality traits during childhood can generate a vulnerability to develop eating disorders, such as anorexia nervosa (AN) or bulimia nervosa (BN), during adolescence and adulthood [233].

Several studies have investigated the neurobiological aspects of eating disorders, including brain imaging techniques such as functional magnetic resonance imaging (fMRI). Thus, neuroimaging makes it possible to characterize the neuronal vulnerability factors that increase the risk of weight gain and risky eating behaviors [234]. The results of neuroimaging studies indicate that people with eating disorders present structural and functional alterations in the brain regions involved in reward processing, emotion regulation, body image perception, and cognitive control [234]. Specifically, the most common structural abnormalities found were global reductions in the gray matter and white matter in AN and similar findings in BN [235]. These abnormalities may contribute to the development and maintenance of disordered eating behaviors [236]. However, other investigations have reported a normalization in the volume of gray and white matter in people who have recovered from these pathologies [237].

In addition, it has recently been postulated that certain alterations in the reward mechanisms of the brain could be involved in the pathophysiology of eating disorders [234]. This process is known as anhedonia, which involves a decreased ability of the person to experience the reward. Continuing with the reward system, the visualization of food activates a series of anticipatory responses that condition each person’s eating behavior [238]. However, people suffering from these EDs have been shown to have poor visual processing of food [234]. An interesting study carried out by Uher et al. [239], in which the visual response of women with anorexia and bulimia is compared, reported a similar activation of the medial prefrontal cortex and inhibition of the lateral prefrontal cortex.

Another important factor that can increase the neuro-vulnerability of people suffering from eating disorders is their interpretation of body image, usually overestimating it, as well as their body weight. This fact has some repercussions on the activation of brain regions. In this sense, Fladung et al. [240] reported activation of the ventral striatal zone, which is associated with emotional processing, by showing photos of underweight women to patients with AN. This is very interesting since it is possible that the comparison of their body with idealized female bodies elicits reward behavior in patients with eating disorders [234]. Furthermore, excessive physical activity is usually a practice carried out by people who suffer from these disorders since it has been postulated that it helps them reduce anxiety caused by food consumption [241]. In this line, Kullmann et al. [242] observed an increased prefrontal cortex in AN patients after visualizing exercise images in comparison with athletes and non-athletes. As with the visualization of food, the visualization of people undertaking physical exercise can stimulate this reward effect at the brain level in these patients.

The exact relationship between neural vulnerability and eating disorders is complex and not yet fully understood. However, it is believed that genetic, environmental, and even gender factors may contribute to the susceptibility of certain people to developing eating disorders. Thus, it is known that women are at greater risk than men of suffering from eating disorders; this difference increases in adolescence [243]. In this sense, the main factor responsible for this is the difference in sex steroid hormones between both sexes. Specifically, the variability of the risk between sexes and within the sex of those suffering from some type of eating disorder could respond to the modulation of hormones to the neural response of the signals related to eating [244].

Despite all, further research is needed to better understand the specific neuronal vulnerabilities and mechanisms underlying eating disorders. This knowledge could potentially lead to improved diagnostic tools and targeted treatments for the individuals affected by these conditions.

## 10. Neuro-Vulnerability and Obesity

Obesity is a complex condition that is influenced by a variety of factors, including genetic, environmental, and behavioral factors. Recent research has suggested that neural vulnerability factors may also play a role in the development of obesity. These factors are thought to increase the risk of overeating and weight gain by altering the brain’s function and structure [245]. It was found that neurobehavioral correlates of obesity are largely heritable, suggesting that genetic factors may contribute to the neural vulnerability factors that increase the risk of obesity [246]. Other research has shown that obesity and a diet high in sugar and saturated fat may lead to brain changes that hamper impulse control, decision-making, and memory [247]. Brain imaging studies have also shown structural changes in the brains of obese individuals [248]. Once this condition has developed in the subject, it has been demonstrated that whole brain cortical thickness is larger in people who are overweight or obese compared to people who are at a healthy body weight, according to a cross-sectional study involving a diverse sample of ages [249]. There is also a decrease in the structural integrity of the white matter tracts that connect various brain regions. Demyelination of these tissues reduces the anisotropic diffusion of water along the axons, as evidenced by a decrease in fractional anisotropy (FA), which is indicative of a decline in structural integrity as obesity levels rise [250]. In this line, theoretical work has suggested that obesity is related to the enhanced incentive salience of food cues, which may contribute to overeating and weight gain. However, evidence from both behavioral and neuroimaging studies has been mixed, with some studies finding an enhanced neural reactivity to food cues in obese individuals and others finding no differences between obese and non-obese individuals [251].

**Figure 2:**
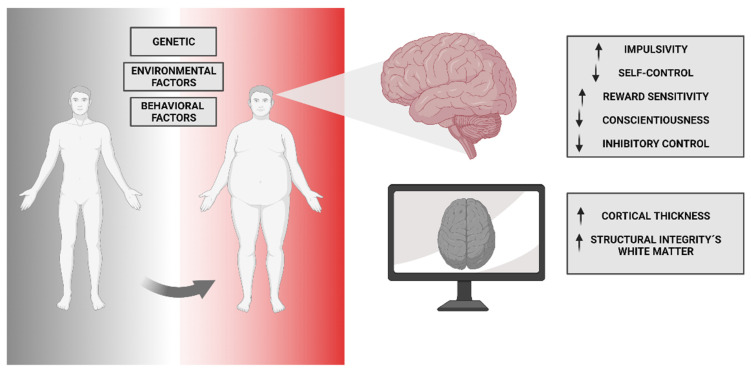
The obesity-related factors that make the obese person vulnerable to various changes at the brain level.

Due to the results in this field being ambiguous, maybe due to the great phenotypic complexity of addictions and obesity, it should be made clear that the tendency to act quickly without fully considering the implications is referred to as impulsivity [252]. The feature is assumed to be the outcome of a combination of strong arousal responses to possible rewards and poor self-control. According to research, impulsivity makes people more vulnerable to addiction and fat. In this regard, lower self-control, reward sensitivity, and negative affect are three impulsivity-related domains that are particularly relevant for the understanding of the similarities between addiction and obesity (Figure 2) [253]. Individual differences in the neurobiological systems involved in the management of food choices and food intake, on the other hand, are likely to explain why some people are more prone to weight gain than others. Individuals with frequent disinhibited behavior and a heightened sensitivity to possible rewards, for example, may be more sensitive to developing unhealthy weight gain when exposed to an “obesogenic” food-abundant environment [246,254]. Likewise, individual impulsive differences may be a common denominator in obesity and drug addiction. Several research studies have revealed that there are similarities in reward processing between addiction and fat [255].

Regarding personality traits, the high disinhibition and low conscientiousness domains of impulsivity are consistently related to a higher risk of addiction, highlighting the need for self-control in avoiding drug misuse [256]. Similarly, obesity has been repeatedly linked to a lower level of conscientiousness [257]. In relation to this, Meule and Blechert discovered that higher attentional and motor impulsivities were predictive of a higher body mass index (BMI) after statistically adjusting for age and gender in a large heterogeneous population. Nevertheless, the effect was minor, and non-planning impulsivity was not found to be significantly related to BMI [258]. However, Hays et al. reported that higher levels of habitual disinhibition, as measured by the Three-Factor Eating Questionnaire, have been linked to long-term body weight growth [259]. Even so, the data remain inconsistent, as a more recent study found no changes between groups in the ability to regulate their impulsive responses [198]. Despite this, disinhibition is an awareness and self-control-related attribute that refers to the tendency to overeat when exposed to appetizing foods or stressful situations. Studies ongoing suggest that obesity is associated with high disinhibition and low conscientiousness. These characteristics may increase a person’s propensity to consume in specific situations and make it more challenging for obese individuals to maintain weight loss behaviors [260]. Unlike conscientiousness and disinhibition, the link between neuroticism/negativity in the emotional domain and being overweight is not quite as strong. Adiposity is not guaranteed; however, this personality feature may make a person more prone to binge eating when they are feeling down [261].

Another impulsivity domain is the concept of risk sensitivity. It provides an additional perspective on impetuous decision-making. Risk sensitivity refers to the degree to which an individual is attracted to or repelled by uncertain outcomes [262]. It has been used to define impulsive behavior in addiction and obesity in recent years [263]. Likewise, obesity has been associated with weak inhibitory control. Obese and overweight individuals have inferior inhibitory control performance on food-specific versions of the stop-signal task, according to a comprehensive literature review [264]. For instance, Hall et al. [265] discovered that people with greater degrees of inattention tended to consume more high-calorie snacks. Also, the conventional Stroop test is associated with lower scores in the obese population, as was shown in a recent study [266].

The functional and structural neural correlates of drug abuse and obesity susceptibility have been studied using neuroimaging. Addicts’ brains appear to respond to drug signals with heightened activity in the ventral striatum, amygdala, and medial regions of the orbitofrontal cortex, as measured by functional magnetic resonance imaging [267]. Enhanced food reward sensitivity (incentive salience of the cue) and poor inhibitory control may contribute to susceptibility to weight gain and overeating, as suggested by numerous brain imaging studies. The anterior cingulate cortex (ACC), dorsomedial prefrontal cortex (PFC), ventral striatum, para hippocampal gyrus, precentral gyrus, superior/inferior frontal gyrus (IFG), and insula are all more active in obese people than in lean people in response to visual food stimuli. Then, evidence from studies shows that there are common brain processes between obesity and various addictions. Both obesity and addiction illnesses may be associated with impairments in inhibitory control, as well as elevated reward sensitivity and heightened attention to signals (foods or substances) [268]. However, despite the similarities highlighted, there is evidence that obesity and other addictive behaviors are distinct and may only partially overlap [269]. While some studies have found a higher prevalence of addictive disorders in obese populations [270], others have found no correlation between obesity and addiction [271]. Due to this, methodological considerations and the extraordinary intrinsic complexity and heterogeneity of obesity and addiction may help to explain the discrepancies between the studies in understanding the level of neuro-vulnerability experienced by obese and overweight people.

In summary, obesity has been linked to personality modifications, brain structure, and cognitive impairment; however, the processes by which this is achieved remain unclear. Current research suggests that insulin resistance, inflammation, and vascular dysfunction may all play a role. Cognitive impairment due to obesity may be reversible, and there is evidence to suggest that weight loss is linked to improved brain and cognitive results [272]. Psychodynamic (insight-oriented) psychotherapy is helpful in assisting with conflicts regarding excessive weight, body image, relationship to food and disordered eating patterns, and dealing with prejudice and overt discrimination that obese patients may experience [273].

## 11. Therapeutic Strategies for Modulating Neuro-Vulnerability

Therapeutic strategies for modulating neuro-vulnerability in obesity have been proposed as a potential avenue for preventing and treating obesity. Several psychological treatments have been found to be effective for binge-eating disorder, including cognitive-behavioral therapy (CBT), dialectical behavior therapy (DBT), and behavioral weight loss (BWL) [274]. Research has also identified several neural vulnerability factors that may increase the risk of overeating and weight gain, including emotion reactivity, food cue reactivity, and craving [275]. These factors may be targeted through interventions such as mindfulness-based stress reduction (MBSR), which has been found to reduce food cravings and improve self-regulation in obese individuals [276]. Other potential therapeutic strategies for modulating neuro-vulnerability in obesity include pharmacological interventions and brain stimulation techniques, such as transcranial magnetic stimulation (TMS) and deep brain stimulation (DBS) [277,278]. These interventions have shown promise in reducing food cravings and improving self-regulation in obese individuals, although more research is needed to determine their efficacy and safety. Therapeutic strategies for modulating neuro-vulnerability in obesity are an emerging area of research that holds promise for preventing and treating obesity. Psychological treatments, such as CBT, DBT, and BWL, as well as interventions, such as MBSR, TMS, and DBS, may be effective for targeting the neural vulnerability factors that contribute to overeating and weight gain.

Recent studies have investigated the potential of transcranial direct current stimulation (tDCS) as a therapeutic strategy for modulating neuro-vulnerability in obesity. It was found that tDCS may be effective in reducing food cravings and food intake in individuals affected by obesity and overweight [279]. Other studies have investigated the potential of tDCS to improve lower limb motor performance in healthy adults and people with strokes [280], as well as its effects on self-paced exercise performance and electroencephalographic (EEG) oscillatory brain activity in trained male cyclists [281]. Research has also identified several neural vulnerability factors that may increase the risk of overeating and weight gain, including emotion reactivity, food cue reactivity, and cravings [282]. In this line, neurobehavioral correlates of obesity are largely heritable, suggesting that genetic factors may contribute to the neural vulnerability factors that increase the risk of obesity [246]. Other research has shown that being overweight or obese and having a diet high in sugar and saturated fat may lead to brain changes that hamper impulse control, decision-making, and memory [283].

Other research has shown that food reinforcement and delay discounting are two factors that may contribute to obesity. Food reinforcement refers to the degree to which a person is motivated to eat by the pleasure or reward associated with food, while delay discounting refers to the tendency to choose immediate rewards over delayed rewards, even if the delayed rewards are larger [284]. Studies have found that food reinforcement and delay discounting interact to predict energy intake and that delay discounting moderates the effect of food reinforcement on energy intake among non-obese women [285,286]. These findings suggest that individuals who are highly motivated by the pleasure or reward associated with food and who tend to choose immediate rewards over delayed rewards may be at increased risk for overeating and weight gain. One study found that food reinforcement, delay discounting, and dietary disinhibition were all predictive of weight gain in non-obese adults [287]. The study suggests that these factors may contribute to the development of obesity by promoting overeating and a lack of self-control around food. Overall, research suggests that food reinforcement and delay discounting may be important factors to consider in the prevention and treatment of obesity. Interventions that target these factors, such as mindfulness-based stress reduction (MBSR) and cognitive-behavioral therapy (CBT), may be effective in reducing overeating and promoting healthy eating behaviors.

The significance of multidisciplinary interventions in modulating the psychophysiological response of patients cannot be overstated [288,289,290]. By bringing together professionals from various disciplines, such as psychiatrists, psychologists, neurologists, and therapists, a comprehensive and holistic approach can be adopted to address the complex interplay between mental and physical health [291,292,293,294,295]. This collaborative effort allows for a more nuanced understanding of the phylogenetic and behavioral bases of the disease, encompassing both genetic predispositions and environmental factors that contribute to its development [296,297]. By gaining insights into these underlying mechanisms, tailored interventions can be implemented to target neuro-vulnerability and promote better outcomes [298,299]. The integration of multiple perspectives and expertise enables a more comprehensive assessment, personalized treatment plans, and improved patient care in the field of mental health [300,301].

## 12. Future Directions and Research Gaps

While significant progress has been made in understanding the neuro-vulnerability associated with energy metabolism regulation, several important research directions and gaps remain to be addressed:

- Longitudinal Studies: Conducting longitudinal studies that track individuals over time can provide valuable insights into the temporal dynamics of neuro-vulnerability and its association with metabolic dysregulation. This approach can help identify early markers or predictors of neuro-vulnerability and inform targeted interventions.
- Genetic and Epigenetic Influences: Investigating the genetic and epigenetic factors underlying neuro-vulnerability can enhance our understanding of individual differences in energy metabolism regulation. Identifying specific genetic variants or epigenetic modifications associated with neuro-vulnerability may open avenues for personalized interventions and precision medicine approaches.
- Gut–Brain Axis: A further exploration of the bidirectional communication between the gut and the brain, particularly through the gut–brain axis, is crucial for unraveling the mechanisms involved in energy metabolism regulation. Understanding how gut-derived signals modulate the neural circuits involved in metabolic control can pave the way for novel therapeutic strategies.
- Sex Differences: Examining potential sex differences in neuro-vulnerability and metabolic dysregulation is important, as sex hormones and other sex-specific factors may contribute to variations in energy metabolism regulation. Investigating whether neuro-vulnerability manifests differently between males and females could guide the development of sex-specific interventions.
- Neuroinflammation and Metabolism: Elucidating the intricate relationship between neuroinflammation and metabolic health is an area of ongoing investigation. Identifying the underlying mechanisms linking neuroinflammation, neuro-vulnerability, and metabolic dysregulation can provide insights into novel therapeutic targets for managing metabolic disorders.
- Advanced Neuroimaging Techniques: Utilizing advanced neuroimaging techniques, such as functional magnetic resonance imaging (fMRI), positron emission tomography (PET), and magnetic resonance spectroscopy (MRS), can provide more detailed information about neuro-vulnerability and its impact on energy metabolism regulation. These techniques can help elucidate the neural circuits and metabolic pathways involved, allowing for targeted interventions.
- Neuro-Vulnerability and Aging: Investigating the impact of neuro-vulnerability on energy metabolism regulation in the context of aging is an important area for future research. Understanding how age-related changes in the brain and neuroendocrine systems influence metabolic dysregulation can inform strategies for healthy aging and prevent age-related metabolic disorders.
- Novel Therapeutic Approaches: Developing innovative therapeutic approaches that specifically target neuro-vulnerability holds promise for improving metabolic health. Exploring neuroprotective agents, neuromodulation techniques, and precision medicine strategies tailored to individuals with neuro-vulnerability can pave the way for more effective interventions.
- Translational Research: Bridging the gap between basic research and clinical applications is crucial for translating the findings into meaningful interventions. Conducting translational studies that integrate preclinical models, human research, and clinical trials can accelerate the development of targeted therapies for individuals with neuro-vulnerability.
- Socioeconomic Factors: Considering the influence of socioeconomic factors on neuro-vulnerability and metabolic dysregulation is important for addressing health disparities. Investigating how socioeconomic status, access to healthy food, and environmental factors interact with neuro-vulnerability can inform public health policies and interventions aimed at promoting metabolic health.
- Comorbidities and Multifactorial Approaches: Given the complex nature of metabolic disorders, exploring the interplay between neuro-vulnerability and comorbid conditions (e.g., cardiovascular disease, diabetes) is necessary. Adopting a multifactorial approach that considers the contributions of both neuro-vulnerability and other systemic factors can enhance our understanding of metabolic dysregulation.
- Lifestyle Interventions: Investigating the effects of lifestyle interventions, such as dietary modifications, physical activity, and behavioral interventions, on neuro-vulnerability and metabolic health is crucial. Understanding how lifestyle factors interact with neuro-vulnerability can inform evidence-based recommendations for preventing and managing metabolic disorders.
- Data Sharing and Collaboration: Encouraging data sharing and collaboration among researchers is vital for advancing our knowledge of neuro-vulnerability in energy metabolism regulation. Promoting open science practices and establishing collaborative networks can facilitate the pooling of resources, data, and expertise, leading to more comprehensive and impactful research outcomes.

Addressing these future directions and research gaps will deepen our understanding of neuro-vulnerability in energy metabolism regulation, paving the way for improved diagnostic strategies, targeted interventions, and, ultimately, better metabolic health outcomes.

## 13. Conclusions

In this comprehensive narrative review, we have delved into the intricate topic of neuro-vulnerability in energy metabolism regulation. By examining the neurobiological basis of energy metabolism, neuroendocrine interactions, neural control of food intake and energy expenditure, and the role of neuroinflammation, we have gained valuable insights into the complex mechanisms underlying metabolic dysregulation.

The findings from this review highlight the crucial role of neural circuits and systems in maintaining metabolic homeostasis. Neuro-vulnerability, characterized by dysfunction and dysregulation within these neural pathways, has emerged as a key factor contributing to the development of metabolic disorders, including obesity, diabetes, and eating disorders. Understanding the underlying mechanisms of neuro-vulnerability is essential for the development of targeted interventions and therapeutic strategies.

This review also emphasizes the importance of interdisciplinary research, bridging knowledge from neurobiology, endocrinology, neuroimaging, and related fields. Future research directions should focus on longitudinal studies to unravel the temporal dynamics of neuro-vulnerability and its association with metabolic dysregulation. Exploring genetic and epigenetic influences, investigating the gut–brain axis, considering sex differences, and examining the impact of neuro-vulnerability on aging are important areas that warrant further investigation. The advancement of neuroimaging techniques, coupled with translational research, holds great promise for deepening our understanding of neuro-vulnerability and its implications for metabolic health. By identifying novel therapeutic approaches and considering the multifactorial nature of metabolic disorders, personalized interventions targeting neuro-vulnerability can be developed to improve outcomes.

It is crucial to acknowledge the research gaps and challenges that lie ahead. Addressing the influence of socioeconomic factors, promoting data sharing and collaboration, and adopting a holistic approach that incorporates lifestyle interventions and comorbidities will further enhance our understanding of neuro-vulnerability and its broader implications.

## Data Availability

Not applicable.
